# Long distance laser filamentation using Yb:YAG kHz laser

**DOI:** 10.1038/s41598-023-45660-9

**Published:** 2023-10-29

**Authors:** Pierre Walch, Benoît Mahieu, Victor Moreno, Thomas Produit, Ugo Andral, Yves-Bernard André, Laurent Bizet, Magali Lozano, Clemens Herkommer, Michel Moret, Robert Jung, Robert Bessing, Sandro Klingebiel, Yann Bertho, Thomas Metzger, André Mysyrowicz, Jean-Pierre Wolf, Jérôme Kasparian, Aurélien Houard

**Affiliations:** 1 Laboratoire d’Optique Appliquée, ENSTA Paris, École Polytechnique, CNRS, IP Paris, 828 boulevard des Maréchaux, 91762 Palaiseau, France; 2 Groupe de Physique Appliquée, Université de Genève, Ch. de Pinchat 22, 1211 Geneva 4, Switzerland; 3 TRUMPF Scientific Lasers GmbH + Co. KG, Feringastr. 10a, 85774 Unterföhring, Germany; 4 Université Paris-Saclay, CNRS, Laboratoire FAST, 91405 Orsay, France; 5 André Mysyrowicz Consultants, 6 Rue Gabriel, 78000 Versailles, France; 6 Institute for Environmental Sciences, Université de Genève, Bd Carl Vogt 66, 1211 Geneva 4, Switzerland

**Keywords:** Nonlinear optics, Laser-produced plasmas

## Abstract

In the framework of the Laser Lightning Rod project, whose aim is to show that laser-induced filaments can guide lightning discharges over considerable distances, we study over a distance of 140 m the filaments created by a laser system with J-range pulses of 1 ps duration at 1 kHz repetition rate. We investigate the spatial evolution of the multiple filamentation regime using the fundamental beam at 1030 nm or using combination with the second and third harmonics. The measurements were made using both a collimated beam and a loosely focused beam.

## Introduction

Laser filamentation has been observed in condensed matter only few years after the invention of the laser^1^. The advent of the chirped pulse amplification strongly boosted the peak power delivered by lasers from the megawatt to the gigawatt^2^, and soon to the TW level, allowing the observation of laser filaments in air^3^, where the nonlinear refractive index is three orders of magnitude lower. While in condensed matter filamentation is deemed a detrimental effect causing damage to optical components, in air it opens the way to many atmospheric applications, including the control of electric discharges^4–8^ and lightning^9–11^, THz to microwave generation^12–14^, air lasing^15^, remote sensing^16–18^, optical waveguide^19,20^, fog clearing^21^ or laser-induced condensation^22–25^.

These applications are fueled by the ability of laser filaments to form and propagate at distances up to the kilometer-range^26,27^, forming long, continuous, slightly ionized plasma channels sustained by a dynamic competition between focusing and defocusing effects of different nonlinear orders^28–30^. The resulting intensity clamping, associated to the short pulse duration that drastically limits avalanche ionization, allows the electron density to stay below the critical density and the filaments remain essentially transparent.

In the context of high-voltage discharge and lightning control, the effect of the laser does not only rely on the free charge carriers (electrons) released by ionization. The energy deposited by the ionization creates a shockwave that results in a transient post-pulse depletion of the air density by up to 70%^31–34^, increasing the mean free path of the electrons and reducing their attachment rate to positive ions. This effect, known as Paschen’s law^35^, reduces the breakdown threshold and contributes to the discharges guiding and triggering^36^.

One main limitation of laser-created filaments to trigger lightning over long distance is the limited lifetime of the plasma (in the nanosecond range)^37^, which at the typical propagation velocity of a guided leader ($10^6$ m s^-1^)^6,38^, limits the effect of the free electrons to the meter-scale. The air-depleted channel lasts longer, with a decay over several hundred microseconds, and a remaining of a few percent only after one millisecond^34^. However, the nanosecond to microsecond lifetime of the ionized and air-depleted channels produced by laser filaments is considered as the main limitation for atmospheric-scale control of lightning^9^. Attempts to circumvent this limitation by using pulse trains have shown limited efficiency^39,40^ due to limited total pulse energy (several mJ) and repetition rate (10–20 Hz), hence an average power limited to tens of mW to the Watt range. The use of laser pulses in the UV^41,42^, far IR^43^, or of dual laser pulses^44^ or 100 TW laser pulses^45^ has also been studied in laboratory, but these laser sources do not allow generating long-lived extended filaments with the present technology.

However, the recent advent of ultrashort thin-disk technology^46^ allows to simultaneously reach sub-ps pulse duration, Joule-range pulse energy, and a kHz repetition rate^47^. Therefore, a laser with TW-range peak power together with a kW-level average power raises new perspective for long-distance discharge control^11,47^. Beyond a higher ionization due to longer pulses, a kHz repetition rate allows each pulse to propagate in the remaining of the depleted channel left by the previous ones, allowing the build-up of a cumulative effect that is spectacularly illustrated in the case of cloud clearing^21^ and results in the establishment of a quasi-steady-state depleted channel prone to a permanent highly-localized Paschen effect^48,49^.

While filamentation is well-known, including on atmospheric ranges, for 10 Hz Ti:Sa lasers at 800 nm, thin-disk Yb lasers simultaneously feature a slightly higher wavelength, a longer pulse duration and an associated narrower spectrum, and, more important, a kHz repetition rate allowing thermal coupling between successive pulses. They therefore deserve a full characterization in view of atmospheric applications. Here, we characterize the propagation of such high-peak-power, high-average-power pulses over atmospheric-range distances (140 m), in the multiple filamentation regime, for the fundamental wavelength (1030 nm) as well as the second and third harmonics. We show that the onset distance of filaments can be controlled by both chirp and geometrical focusing, and that filament can propagate up to 140 m distance. We also show that the beam tends to self-organize as a filament ring unless forced into a superfilament by geometrical focusing. Furthermore, the partial frequency-doubling of the pulses perturbs the filamentation of the fundamental wavelength through coupling effect between the two colors. We finally characterize the air density channel in different focusing conditions.

## Experimental setup

We used the thin-disk Yb:YAG laser source described in^47^, which provides pulses at 1030 nm center wavelength with an energy of 500 mJ after compression and a minimum pulse duration of 1 ps, at a repetition rate of 1 kHz. The laser output beam has a beam diameter of about 30 mm (cross section of 700 mm^-2^), with an average $M^2$ value of 2.1. The use of lithium triborate (LBO) crystals enables us to produce 295 mJ (59% conversion efficiency) and 119 mJ (27% conversion efficiency) at respectively the second (515 nm) and the third harmonic (343 nm)^50^. A specially-designed telescope can expand the beam by a factor of 7.14 and focus it at a distance ranging from 55 m to infinity^10^. In our measurement, the propagation distance *z* along the laser axis is defined as the distance between the output of the telescope and the diagnostics. Our experimental setup was installed in a 150 m long hall at the University Paris-Saclay (Orsay, France).

To characterize the presence of filaments along the laser path, beam impact measurements were realized every meter along the laser propagation using photosensitive paper (Ilford Multigrade IV RC 44M). The paper was swept sufficiently fast across the beam path to distinguish the impact of individual shots, while the laser was continuous working at 1 kHz. In addition, to quantify the energy deposition in air by the filaments we characterized the acoustic emission along the laser propagation path. During ionization, the filamentary laser pulses quickly deposit energy in the medium resulting in the creation of a lateral pressure wave that can be measured by a microphone^51–53^. The evolution of the acoustic signal along the laser path was measured for the same pulse durations used previously by means of a microphone (model G.R.A.S. 46 HB) amplified by an amplifier (model G.R.A.S. 12AK) with a gain of 40 (detection bandwidth 20 Hz–20 kHz). We considered the integrated magnitude of the absolute value of the signal over 0.2 ms as representative of the pressure wave magnitude.

## Results

### Long distance filamentation using a collimated beam

#### Effect of the pulse duration

We first investigated the filamentation of the free-propagating collimated laser beam. In such a configuration, the control of the filamentation distance can be achieved simply by adjusting the laser pulse duration^54^.

**Figure 1:**
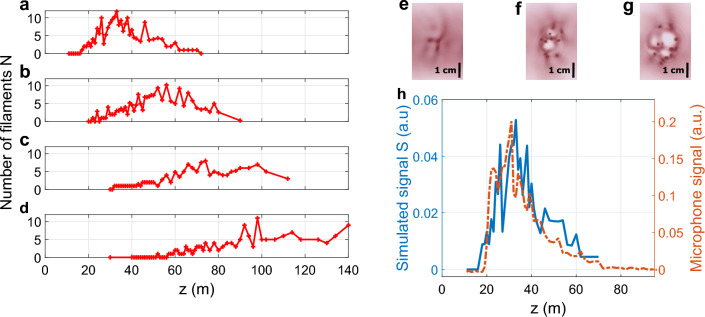
Evolution of the number of filaments *N* along the laser propagation axis *z* of a 500 mJ 1030 nm laser pulse, for pulse durations of (**a**) 1 ps, (**b**) 3 ps, (**c**) 5 ps and (**d**) 7 ps. Examples of photosensitive paper measurements performed at the distances (**e**) *z* = 19 m, (**f**) *z* = 25 m, (**g**) *z* = 29 m for a pulse duration of 1 ps. (**h**) Comparison between the microphone signal (orange dashed line) and the simulated signal *S* (blue solid line) along the laser propagation axis *z* for a laser pulse of 500 mJ at 1030 nm with a duration of 1 ps.

The evolution of the number of filaments *N* as a function of the propagation distance *z* was measured in the case of 1030 nm 500 mJ pulses at several pulse durations ranging from 1 to 7 ps. The results, displayed in Fig. 1, show that filaments are present over several tens of meters for all pulse durations. As expected from previous observations at 800 nm^27,54,55^, and the Marburger formula, longer pulses generate filaments at a longer distance. Indeed, as shown in Table 1, the position of the maximum number of observed filaments matches well the Marburger formula for self-focusing distance $L_c$ applied to the whole beam^56^:

1 $$\begin{aligned} L_c=\frac{0.367\ z_r}{\sqrt{\left( \frac{P_{in}}{P_{cr}}-0.852\right) ^2-0.0219}}, \end{aligned}$$

with $P_{in}$ the initial peak power of the laser pulse, $P_{cr} = 4.9$ GW the critical power for self-focusing in air at 1030 nm^57^ and $z_r$ the Rayleigh length ($z_r = 690$ m at 1030 nm).

Furthermore, the onset of filamentation is close to the local application of the Marburger formula on each filamenting cell within the beam, showing that the nucleation of the observed small scale filamentation is governed by the local splitting of the beam profile into self-focusing cells influenced by inhomogeneities originating from the initial beam profile and from the effect of turbulence in the experimental hall.

Finally, let us note that the filamenting region is also longer for longer pulses (Fig. 1), although the distance available for the experiment was limited to 140 m, preventing to measure the full length of the filament region for the pulse duration of 7 ps.

**Table 1:** Comparison between the distance at which a maximum of filaments is detected and the whole-beam self-focusing distance $L_c$, for pulse durations of 1 ps, 3 ps, 5 ps and 7 ps.

Laser pulse duration (ps)	1	3	5	7
Peak power (GW)	500	167	100	71
Position of the peak of filament number (m)	33	56	74	98
Calculated filamentation distance $L_c$ (m)	30	56	76	94
Length of filamentation region (m)	50	70	80	> 90

We compared the measured acoustic signal with a simulated signal based on the sum of the pressure waves emitted by all individual filaments detected with the photosensitive paper, each one being located at a different distance from the microphone. This propagation-corrected simulated signal *S*, can be expressed as

2 $$\begin{aligned} S=\frac{CN}{2\pi R^2} \int _{0}^{2\pi }\int _{0}^{R}\frac{r\ dr\ d\theta }{\left( D-r\cos \theta \right) ^2+\left( r\sin \theta \right) ^2}, \end{aligned}$$

where *r* and $\theta$ are the cylindrical coordinates, *R* the radius of the filamentation zone, *D* the distance between the center of the beam and the microphone, *N* the number of filaments and *C* is the signal emitted by a single filament. The integral corresponds to the averaging over a homogeneous probability distribution of filaments within the filamentation zone, considering 1000 shots generating independent filament patterns. Since distances between filaments are in the cm-range, well shorter than the acoustic wavelength, we integrate the sound intensity rather than the signal amplitudes. The simulated signal *S* is calculated using the values of *R* and *N* measured from the beam characterization with the photosensitive paper.

The comparison between the experimental acoustic signal and the simulated signal calculated from the photosensitive paper measurements for a laser pulse of 500 mJ at 1030 nm with a duration of 1 ps is shown in Fig. 1h. The clear proportionality between the two signals confirms that the power carried and deposited by each filament is constant over the propagation distance. The same observation is made for all tested pulse durations.

#### Multi-color filamentation

We used the frequency doubling LBO crystal to study the filamentation of a dual frequency beam consisting of 285 mJ energy at 515 nm and 215 mJ energy at 1030 nm^50^, by repeating the beam impact measurements. The fundamental laser pulse duration was set to 1 ps. A consequence of the frequency doubling is the modification of the filament diameter $d_{fil}$. The proportionality of $d_{fil}$ with the wavelength is given by^58,59^:

3 $$\begin{aligned} d_{fil}=\frac{1.22\ \lambda }{\sqrt{8n_0n_2I_\text{max}}}, \end{aligned}$$

with $\lambda$ the beam wavelength, $n_0$ the air refractive index, $n_2$ the nonlinear Kerr refractive index and $I_\text{max}= 2\ 10^{13}$ W cm$^{-2}$ the intensity of the laser in the filament^57^. The filament diameter being directly proportional to the wavelength, the filaments of the frequency-doubled beam have half the diameter of the ones from the fundamental beam. The two populations of filaments can thus be distinguished on images of the beam impacts on photosensitive paper.

**Figure 2:**
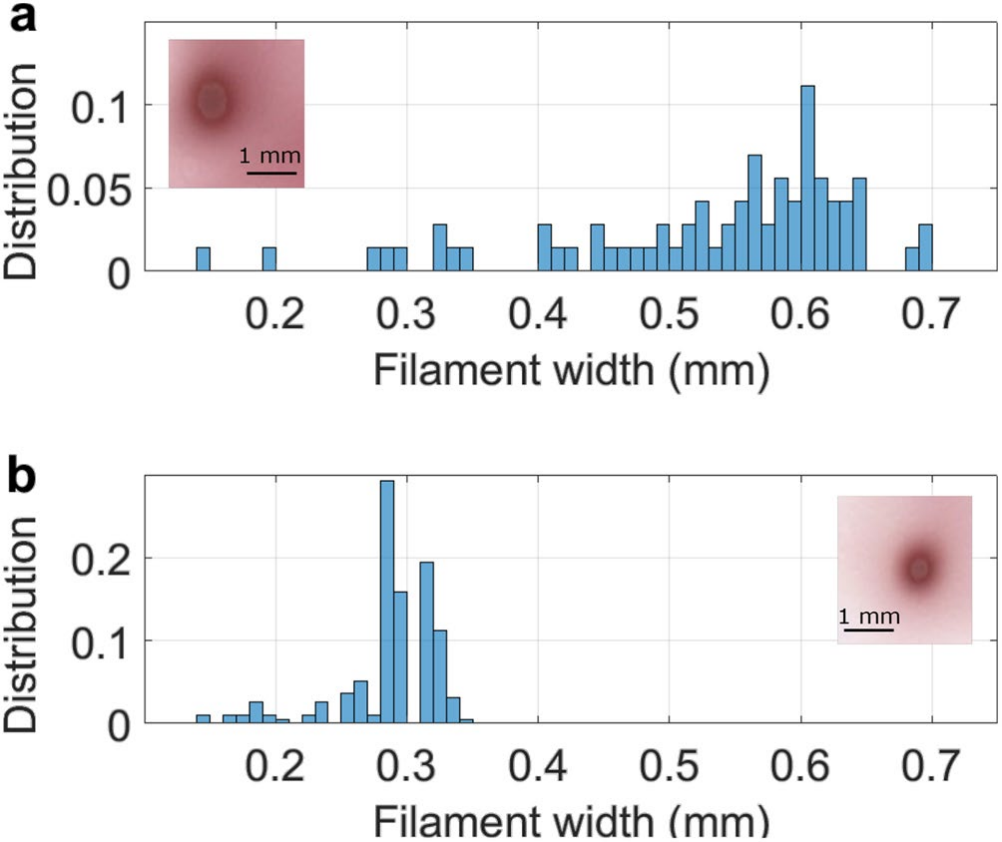
Experimental distribution of the filament widths produced by (**a**) 500 mJ 1 ps laser pulses at 1030 nm and (**b**) 500 mJ 1 ps laser pulses at 1030+515 nm measured on photosensitive paper. Insert: Example of impacts of single filaments on photosensitive paper.

The filament size distributions for the fundamental and dual-color beams indeed display a factor of two in their diameters (Fig. 2). Note that the impacts on the photosensitive paper is typically 5 times larger than the filament diameter reported experimentally and predicted by the theory (120 $\upmu$m and 62 $\upmu$m at 1030 nm and 515 nm, respectively)^29,30^. This is explained by the low threshold of our photosensitive paper that is darkened not only by the filament core but also by the photon bath around it, resulting in a burned mark larger than the filament itself. Remarkably, no filament with a diameter above 0.35 mm (which should be the case for a filament at 1030 nm) is formed while using the dual frequency beam, suggesting that the unconverted fraction of the beam at 1030 nm does not produce filaments despite its 214 mJ energy per pulse ( 43 $P_{cr}$).

**Figure 3:**
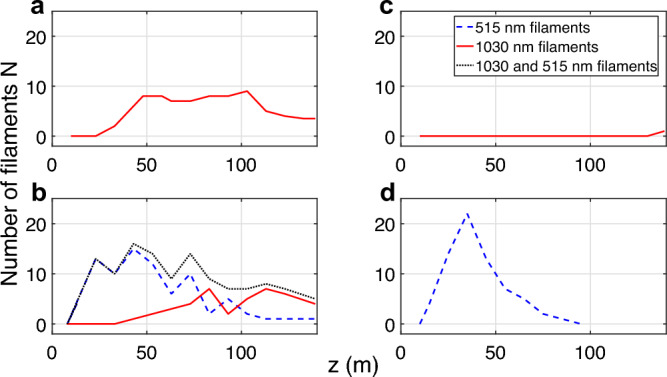
Evolution of the number of filaments *N* at 1030 nm (red solid line) and at 515 nm (green blue dashed line) along the laser propagation, created by (**a**) the fundamental 1030 nm only, (**b**) 1030+515 nm pulses, (**c**) the isolated fundamental part of 1030 + 515 nm pulses and (**d**) the isolated second harmonic part of the 1030 + 515 nm pulses. The dotted line corresponds to the total number of filaments at 1030 nm and 515 nm.

To confirm and understand the latter observation, additional measurements were made. Due to technical issues, the laser was repaired between these two sets of measurements and the beam profile was heavily modified, hence comparison should only be done within the same measurement set. To characterize the filamentation of each wavelength, we measured the number of filaments created by the 1030 nm and by the 515 nm beam by identifying them from impact beam measurements. Filaments up to a diameter of 0.4 mm are identified as 515 nm filaments, while any filament above this diameter is deemed a 1030 nm filament. Four configurations were used: the fundamental beam, the dual-frequency beam, and the individual fundamental and second harmonic beams separated from the dual-frequency one by means of a dichroic beam splitter. The results are presented in Fig. 3. The depletion of the fundamental by the frequency doubling results in a much weaker and later filamentation (compare panels a and c). This difference is mainly explained by the fact that the non-linear depletion of the pump beam tends to self-regularize the beam, i.e., smooth the gradients within the beam profile due to higher conversion efficiency (hence higher losses) in the most intense regions. Such self-regularization is expected to hinder filamentation by reducing the seeding of spatial modulational instability. Modeling this effect based on the depleted pump formalism^60^, in our conditions featuring  57% conversion efficiency into the SHG^50^, shows that the gradients are reduced by a factor of $\sqrt{2}$.

On the other hand, frequency-doubling the beam (compare panels a and d) results in more than twice as many filaments, with an earlier onset. This increase is in line with the fact that the SHG conversion efficiency is beyond 50%, which, together with the $\lambda ^2$ dependence of the critical power^29^ results in more than doubling the normalized power $P/P_{cr}$. More specifically, the critical power at 1030 nm amounts to 4.9 GW, so that 500 mJ in 1 ps corresponds to $100 P_{cr}$. At a rate of 1 filament per $5 P_{cr}$^61,62^, we can therefore expect up to  20 filaments, about twice the number observed. The 12 filaments observed correspond to  $8P_{cr}$/filament. In the case of the frequency-doubled beam at 515 nm, the critical power amounts to 1.24 GW, so that the 285 mJ, corresponding to 285 GW peak power if we assume a 1-ps pulse duration, corresponds to $230 P_{cr}$. The 26 filaments observed correspond to approximately $9P_{cr}$ / filament, again slightly above the ratio expected from measurements for shorter pulses. The beam diameter of 30 mm has a cross section of 700 mm$^2$, corresponding to more than 26 mm$^2$/filament. This value lies far from the geometrical saturation, which amounts to only a few mm$^2$/filament^63^. Therefore, spatial congestion is not the factor limiting the number of filaments.

Finally comparing the dual-color beam (panel b) with the individual colors (panels c and d) evidences a nonlinear interaction between the two frequencies during the propagation. This interaction (1) reduces the total number of filaments (compare panel b with panels c and d), (2) increases the total filamenting length, (3) restores / anticipates the filamentation of the remaining fundamental.

One other important observation is that the isolated fundamental part and second harmonic part of the dual frequency beam show a different filamentation process when they are isolated from each other, as seen in Fig. 3c,d. When isolated, the fundamental part of the beam only starts filamentation after a propagation of more than 135 m (as compared to 40 m in the dual frequency beam). This can be explained by the regularization effect and the reduction of the available power induced by the SHG crystal on the fundamental beam. On the other hand, the isolated second harmonic part of the beam starts the filamentation process after the same propagation distance of 13 m but the maximum number of filaments is higher and their spread is limited to a smaller distance compared to what is measured when using the dual frequency beam. These two observations suggest that the two parts of the beam interact during the propagation and improve the filamentation of the fundamental part of the beam while hindering the filamentation of the second harmonic part. It is important to observe that, due to a phase velocity mismatch in air of 46.5 fs m$^{-1}$ between these two spectral components, they do not temporally overlap anymore once the filament process begins after about 20 m of propagation, so the optical interaction of the two parts of the beam only occurs over this distance. But they can also interact through the ionization or the molecular orientation left by the first IR pulse, or through cumulative thermal effects that persists over very long timescale.

Another aspect of the beam affected by the nonlinear propagation is its spectrum. The high beam intensity preventing direct measurement of its spectrum, we characterized the laser spectrum at different distances by measuring with a spectrometer the spectrum of the light scattered on a ceramic plate placed on the beam path, using the triple frequency-converted pulse at 1 ps^50^.

**Figure 4:**
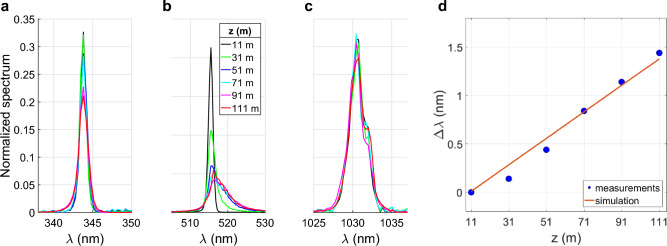
Evolution of the spectrum of the triple frequency-converted pulse along its propagation. (**a**–**c**) being the spectral components around 343, 515 and 1030 nm, respectively. (**d**) Comparison between the measured and calculated redshift along the laser propagation, for the pulse spectral component at 515 nm.

The results, presented in Fig. 4a–c show that the spectral broadening of the mixed beam is much more significant for the second harmonic than for the fundamental and the third harmonic. In the second harmonic component, the spectral width rises from 1.0 to 7.6 nm, which would allow the Fourier-limited pulse durations to drop from 389 to 51 fs. Note that the broadening we observe occurs during the propagation, well after the SHG crystal. The broadening during the propagation before and in the crystal is already taken as an initial condition.

The redshift of the 515 nm beam can be explained by the retarded Kerr effect in air^64^ also referred as Stimulated Raman Scattering^42,65^. Based on the model presented in^27^, the red shift $\Delta \lambda$ is calculated along the laser propagation using the equation:

4 $$\begin{aligned} \Delta \lambda (z)=\lambda _0 \frac{n_2I_0}{\tau \sqrt{2}c} \frac{\Delta t_0}{\Delta t}(z-z_0), \end{aligned}$$

with $\lambda _0$ the initial wavelength, $n_2$ the instantaneous nonlinear index of air, $I_0$ the laser intensity, $\tau$ the characteristic time of the partial alignment of the molecules (1.5 ps), *c* the speed of light, $\Delta t_0$ the minimum pulse duration, $\Delta t$ the pulse duration, *z* the distance of propagation and $z_0$ the onset position of the filament. The calculated values, presented in Fig. 4d, show a good agreement between the theory and the measurement.

The same measurement was repeated for pulse durations of 1 ps, 3 ps and 5 ps. For all tested pulse durations, the spectral broadening of the mixed beam is more important for the second harmonic than for the fundamental and the third harmonic. Furthermore, longer pulse durations delay the onset of the spectral broadening and decrease its importance. The distances where the spectral broadening starts are consistent with the distances of onset of filaments for all pulse durations. Moreover, the broadening only takes place over a limited distance corresponding to the filamentation range. Beyond, no further evolution of the spectrum is observed.

### Long distance filamentation using a focused beam

In a second time, we studied the filamentation induced after our large-aperture focusing telescope (see description in^10^). This configuration is more relevant for applications such as the laser lightning rod^11^ because it allows a control over the collapse distance as well as stronger energy deposition through superfilamentation regime^66^.

#### Filamentation for different focusing distances

By changing the focal length of the telescope, we studied the filamentation process when the beam is focused at 55 m (Numerical Aperture = 0.0023) or 115 m (N.A. = 0.0011). As for the collimated beam, beam impact on photosensitive paper were used to characterize the presence of filaments along the laser path. However, in this configuration individual filaments cannot be distinguished because they are squeezed by the overall focusing of the beam. Therefore, the total surface occupied by filaments is used as a proxy for to the number of filaments. This measurement was repeated for the two focusing distances using the fundamental and the dual-color beams, for pulse durations varying from 1 to 7 ps.

**Figure 5:**
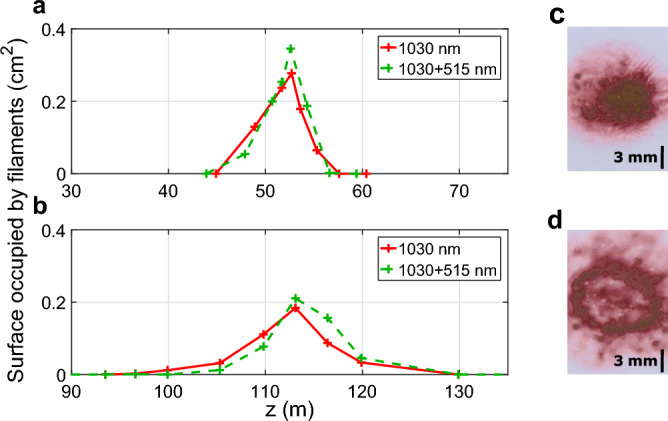
Spatial evolution of the surface occupied by filaments created by the 500 mJ 1 ps fundamental beam (red solid line) or the frequency doubled beam (green dashed line), focused at (**a**) $z=55$ m or (**b**) $z=115$ m. Examples of photosensitive paper measurements performed at the respective focal point (**c**) *z* = 55 m and (**d**) *z* = 115 m.

The results, presented in Fig. 5, show that the use of the frequency doubled beam leads to a higher surface occupied by filaments compared to the fundamental beam, albeit the difference is small. Furthermore, only small differences were observed between measurements at different pulse durations. This is an important observation because it allows us to use long pulse duration to reduce the laser intensity and prevent damage on the optics of our setup while maintaining a high number of filaments. On the contrary, an important difference is observed when comparing the two focusing distances. The longer focusing distance of 115 m results in a lower maximum of the surface occupied by filaments but in a longer filamentation length (defined as the length over which at least one filament is detected), of more than 30 m compared to 12 m for the focusing distance of 55 m. We also note the apparition of a ring like organization of the filaments in the case where the beam is focused at 115 m, while a dense pack of filaments is observed with the focusing distance of 55 m as illustrated in Fig. 5c,d. The latter can be interpreted as a superfilamentation regime^66^.

In addition, we characterized the low-density air channel resulting from the energy deposition by the filamentation process^34,67^. By measuring the phase shift induced by this density depletion on a transverse probe beam (see^48^ for a description of the setup), we characterized the low-density channel present at the focal point for the two focusing distances at $z=55$ m and $z=115$ m, using the fundamental and frequency doubled beam. The measurement was performed after the shockwave has developed, 1 $\upmu$s after the laser pulse, corresponding to the peak of the air density depletion^34^.

**Figure 6:**
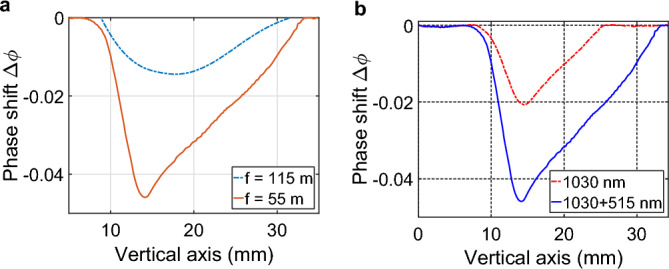
(**a**) Phase shift $\Delta \phi$ induced by the frequency doubled 500 mJ, 1 ps pulse, focused at $z=55$ m (red solid line) and $z=115$ m (blue solid line), measured at the geometrical focus of the beam 1 $\upmu$s after the last laser pulse. (**b**) Phase shift $\Delta \phi$ induced by the 500 mJ, 1 ps frequency doubled beam (blue solid line) and the 500 mJ, 1 ps fundamental beam (red dashed line), focused at $z=55$ m, measured at the focal distance 1 $\upmu$s after the last laser pulse.

As seen in Fig. 6a, we observe an asymmetric phase shift, characteristic of filamentation at high repetition rate^48^. This shape is due to the accumulation of successive low-density channels going progressively upward due to Archimedes thrust. But the transverse size of this phase shift ( 10–15 mm) is significantly larger than the one produced by a single filament (100 $\upmu$m FWHM) or by a kHz laser at 800 nm with an energy of 2 mJ (3 mm FWHM). We also note that the measured phase shift is more important when the beam is focused at $z=55$ m compared to $z=110$ m, as expected since the transversely integrated density of deposited energy increases with the laser numerical aperture. This observation is consistent with the previously measured higher maximum of surface occupied by the filament, when the beam is focused at 55 m. However, while the previous measurements of the occupied surface have shown only minor differences between the fundamental and frequency doubled beam, the measured phase shifts $\Delta \phi$ show an important increase when using the frequency doubled beam, as seen in Fig. 6b. This suggests that while the number of filaments is not higher when using the frequency doubled beam, each filament deposits more energy that result in a greater depletion of the air density.

## Conclusion

We have characterized the filamentation of a 500 mJ, 1 ps, 1 kHz laser system in different focusing conditions. Using a collimated beam, the presence of filaments over 50 m has been observed and the onset of the filamentation was shifted from 18 to 60 m by changing the pulse duration from 1 to 7 ps. Using a large-aperture focusing telescope, we characterized the filaments created by the beam focused at 55 m and 115 m. Filaments created at 55 m are more locally dense than the ones created at 115 m, but they spread over a distance of 12 m compared to the 30 m covered by the ones created at 115 m.

We characterized the low-density air channel resulting from the filamentation and have shown that the one resulting from a tighter focus is more important. The use of frequency doubling crystal also resulted in a more important reduction of the air density.

These measurements were crucial for the main experiment of the Laser Lightning Rod project^10,11^ where this laser system was used to guide ascending lightning leader starting at the top of a tower. Our ability to create filaments at a distance of 115 m was a requirement as this is the tower height, and the measured presence of filaments over 30 m would be an important parameter to scale the guiding effect.

## Data Availability

The datasets generated during and/or analyzed during the current study are available from the corresponding author on reasonable request.
